# Long-Term Followup of Patients with Active J-Reservoirs after Restorative Proctocolectomy for Ulcerative Colitis with regard to Reservoir Function, Mucosal Changes, and Quality of Life

**DOI:** 10.5402/2011/430171

**Published:** 2011-07-16

**Authors:** Ola Røkke, Knut Iversen, Torill Olsen, Sølvi-May Ristesund, Geir Egil Eide, Gitta Erika Turowski

**Affiliations:** ^1^Department of Gastrointestinal Surgery, Akershus University Hospital, 1478 Lørenskog, Norway; ^2^Faculty of Medicine, University of Oslo, 0316 Oslo, Norway; ^3^Department of Surgery, Diakonissehjemmet Hospital, Haraldsplass, 5021 Bergen, Norway; ^4^Department of Surgery, Haukeland University Hospital, 5021 Bergen, Norway; ^5^Centre for Clinical Research, Haukeland University Hospital, 5021 Bergen, Norway; ^6^Research Group on Lifestyle Epidemiology, Department of Public Health and Primary Health Care, University of Bergen, 5020 Bergen, Norway; ^7^Department of Pathology, Ullevaal University Hospital, 0424 Oslo, Norway

## Abstract

*Objective*. Study the functional results and mucosal changes in the ileal pouch after restorative proctocolectomy with J-reservoir for ulcerative colitis. *Material and Methods*. Followup study of 125 patients with J-reservoir with one disease-specific- and one general (SF-36) quality of life-questionnaire, rectoscopy with biopsies, and stool samples to evaluate inflammation, dysplasia, presence of *Helicobacter pylori* and calprotectin level. *Results*. Fourteen J-reservoirs were removed or deactivated, leaving 111 patients for followup. The followup time was 6.8 (1–15) years. 87.4% of the patients were satisfied. 93.1% had some kind of functional restriction: food- (75.5%), social- (28.9%), physical- (37%) or sexual restriction (15.3%). 18.6% had often or sometimes faecal incontinence. Low daytime faecal frequency was associated with good quality of life. 13 patients (12.6%) had a less favourable result. There was no pouch-dysplasia. Calprotectin levels were increased in patients with visible pouch inflammation or history of pouchitis. HP was diagnosed by RUT in 42.3%, but was not associated with inflammation or pouchitis. *Conclusions*. Most patients were satisfied with the J-reservoir in spite of a high frequency of various restrictions. 12.6% (13 patients) had a less favourable functional result, partly due to a high frequency of defecations, pain, pouchitis and inflammation.

## 1. Introduction

Surgical treatment of ulcerative colitis may be indicated when medical treatment fails. Restorative proctocolectomy with ileal pouch-anal anatomosis (IPAA) was introduced in 1978 by Parks and Nicholls [1] and is the most commonly used surgical technique. With a successful functional result, these patients avoid a permanent ileostomy and may lead a normal life.Overall patient satisfaction rates of 90–95% have been reported [2, 3], with a quality of life about the same as in patients with ulcerative colitis in remission [4]. However, several series report on postoperative morbidity rates of 30–50% and long-term failure rates of 10% [5–8]. There is a clinical observation that the quality of life in patients with reservoirs constitutes a spectrum of functional results. The majority of the patients are satisfied, but a significant minority of these patients has complaints and problems that influence their daily life, with lower quality of life than patients with successful reservoirs. There has also been a concern about the risk of dysplasia and malignancy over time from residual rectal mucosa or from the pouch itself, as has been previously reported [9]. 

Even if the tolerance among patients may vary, pain, discomfort, and high faecal frequency will often lead to a reduced quality of life. The choice of reservoir type, the surgical technique during construction, and early and late complications like pouchitis may contribute to this. However, in some patients, the reason for inferior functional result is still unknown. The cause of inflammation, and in a minority of patients dysplasia, is also unknown, even if a microbial cause is suspected [10]. Among the microbial species in the gastrointestinal tract, *Helicobacter pylori* infection is known to cause inflammation and dysplasia in the gastric mucosa and has emerged as a carcinogenic agent also in extragastric malignancies like colorectal and hepatic cancers [11]. 

The aim of the present study was to define the long-term quality of life in the patients with functional J-reservoirs and to evaluate what part of the patient's functional spectrum was especially affected. We also wanted to identify possible factors associated with good and less satisfactorily functional results, including macro- and microscopical characteristics of the reservoir and mucosa as well as factors in the stool-like calprotectin. Finally, we wanted to investigate the presence of *Helicobacter pylori* (HP) and a possible relation between HP and microscopical changes in the pouch mucosa.

## 2. Materials and Methods

A total of 125 consecutive patients with ulcerative colitis treated with restorative proctocolectomy and ileal pouch with the J-reservoir construction [12] from 1988 until 2002 were included in the study. In two of these patients, surgery was indicated due to dysplasia in the colon mucosa. Three others had developed adenocarcinoma. In the other 120 patients, the indication for surgery was benign ulcerative colitis not controlled by medication. Of the 125 patients enrolled in the study, 14 patients were excluded due to postoperative excision of the reservoir (*n* = 1) or delayed reservoir failure with removal of the reservoir (*n* = 8) or defunctioning with loop ileostomy (*n* = 5), leaving 111 patients for followup. 

The J-reservoirs were created by suture machines (3 GIA 80) and were at least 15 cm long (15 to 20 cm). All anastomoses were created with double-stapling technique. The rectal dissection was initially performed close to the rectal wall. After 1995, however, the rectal excision included the mesorectum. A protective ileostomy was used as a routine during the first years, but from 1993, ileostomy was used only when considered necessary by the operating surgeon. 

Medical records were reviewed and recorded in a database (SPSS, Ill, USA). All patients were invited to a followup at the Department of Surgery during 2001-2002 by both a surgeon and a specially trained nurse. Blood samples were drawn for laboratory tests (haemoglobin, leucocytes, C-reactive protein, electrolytes, albumin, creatinine, bilirubin, alkaline phosphatase (ALP), glutamyl-transferase (GT), aminotransferases (SGOT, SGPT), iron, iron-binding capacity, ferritin, cobalamin, and folic acid). Clinical investigation of the perianal area and rectoscopy was performed to evaluate reservoir characteristics. Biopsies were taken from the pouch 5 and 10 cm from the ileorectal anastomoses and from the residual rectal mucosa for microscopical examination. Biopsies were also taken from the pouch for detection of *Helicobacter pylori* microbes by the rapid urease test (RUT). Stool samples were taken for analyses of HP with the immunochromatographic test (ImmunoCard HpSA STAT, Orion Diagnostica) and for analyses of calprotectin (routine laboratory technique, Ullevaal University Hospital, Norway). All patients were asked to answer two written questionnaires that they received by mail prior to the followup: one with specially designed questions about functional status (incontinence, work restriction, social restriction, sexual restriction, and more), the other was the 36-item Short-Form Health Survey questionnaire (SF-36), which records the general quality of life and restrictions in the physical, pain, vitality, social, and mental dimensions [13]. The SF-36 score has been validated in the Norwegian population, and sex- and age-adjusted scores exist for the general population [14]. As many as possible of these questionnaires were reviewed and completed by nurses together with the patient at the followup. Patients neither attending the followup nor responding on the questionnaires received new questionnaires several times to make the study as complete as possible. 

The Norwegian Ethical Committee approved the study.

## 3. Statistics

Numbers in text and tables are given as mean (minimum-maximum) or median (minimum-maximum) when indicated. Student's *t*-test and one-way analysis of variance (ANOVA) was used to test differences between means. Pearson's chi-squared test was used to test differences between groups. The Spearman rank sum test was used to determine significance of correlations.

## 4. Results

There were 65 men (58.6%) and 46 women (41.6%) with mean age at followup 42.6 years (17–72 years). The mean age for start of the disease was 28.0 years (3–60 years), and mean age for reservoir construction was 35.9 years (9–69 years). Three patients (3%) had hypertension (*n* = 1), obstructive lung disease (*n* = 1), and primary sclerosing cholangitis (PSC) (*n* = 1). The others were otherwise healthy. The mean observation time with functional reservoir was 6.8 years (1–15 years). Surgery was performed as either 3-stage procedure in 36 patients (33%), 2-stage procedure in 67 patients (60%), or one-stage procedure in 8 patients (7%). The mean time from start of the disease until reservoir construction was 8 years (1–32 years). The observation time after reservoir construction was 6.8 years (0.8–15.3 years). In this period, three other patients had developed primary sclerosing cholangitis, and one of these had had a liver-transplantation.

### 4.1. Quality of Life Questionnaires

103 patients (92.8%) finally filled in the questionnaires; some, however, with missing values and numbers in the tables and results vary accordingly. Patient characteristics and some elements of the quality of life are summarized in Tables 1, 2. Seventy-nine patients (76%) had a regular relation (married/living together). Eighty-one patients (79.5%) had work in 40 different professions, 68 patients (66.7%) in full-time work, and 12.8% in reduced work. Seven patients (6.9%) were disabled, and 36.6% of patients reported problems often (9.9%) or sometimes (26.7%) with the reservoir in their work, for example, fatigue, frequent toilet visits, in need of access to toilettes, plan business trips, schedules, pain, loss of sleep, and noise from bowel movements. 

 The median number of defecations was at average 6 (2–13) per day and 1 (0–6) per night. Median numbers of defecations during best and worst day- and nighttime periods are given in Table 2. Nineteen patients reported often (5.9%) or sometimes (12.7%) lack of faecal continence. Thirty-eight patients (37.3%) had always complete control. Twenty-six patients always (7.9%) or sometimes (17.8%) used diapers. Forty patients (39.6%) could differentiate between faeces and air. Sixty-five patients (63.7%) used medication for thicker faeces, mostly loperamid, 52 of them (51%) daily. The best, average, and worst faecal frequencies categories at daytime and nighttime were all interrelated with significant correlation as determined with the Spearman correlation coefficient. 

The faecal frequency rates were also related to the SF-36 scores. The patients' best, average, and worst day- and nighttime defecation frequency categories were each divided into three groups of about the same size consisting of patients with the lowest, medium, and highest frequencies in each category. The patients with low faecal frequency had the high SF-36 scores, significantly different in several SF-36-dimensions from patients with high faecal frequency rates. This difference was most clearly demonstrated in patients with high faecal frequency during average and worst daytime periods and best nighttime periods in the physical, bodily pain, general health, and vitality dimensions as shown in Table 3. 

The relation between average faecal frequency and other indicators of quality of life was also significant and is shown in Table 4. Faecal frequency was not associated with age (*P* = 0.377), the use of protective stoma at the reservoir operation (*P* = 0.835), postoperative complications (*P* = 0.455), reoperations (*P* = 0.455), number of pouchites (*P* = 0.158), length of the reservoir (*P* = 0.970), length of the residual rectum (*P* = 0.110), distance of the anastomosis from the anal verge (*P* = 0.070), stenosis in the reservoir (*P* = 0.474), macroscopically signs of inflammation (*P* = 0.858), microscopically inflammation (*P* = 0.858), calprotectin-level (*P* = 0.748), *Helicobacter pylori* test by RUT (*P* = 0.491) or ImmunoCard (*P* = 0.080), or serological parameters (not significant for any serological parameters measured). 

Thirty-three patients (32.4%) used selected food to make their faeces thicker, that consisted of a spectrum of food sorts based on individual experiences, like bananas, bread rich in fibres, fine bread, rice, fruits, vegetables, yoghurt, pasta, cola, dry food, alcohol, and blueberry. Sixty patients (59.4%) avoided special types of food that made their stool thinner, also with a wide spectrum of food sorts like fruits, sweets, chocolate, spicy food, fat food, apples, cabbage, peas, apple juice, kiwi-fruits, nuts, cucumber, shrimp, grapes, watermelon, red wine, corn, and alcohol. Forty-two patients (41.2%) avoided special food sorts because of other problems like abdominal pain, meteorism, constipation, diarrhoea, perianal pain, fever, afraid of bowel obstruction, instant faecal urge, and undigested food in the stool. The food sorts indicated were again a spectrum of sorts like cabbage, fruit peel, apple, orange, asparagus, onion, spicy food, salad, smoked food, shellfish, nuts, pineapple, strawberry, food high in fibres, nuts, salted meat, corn, mushroom, onion, pizza, egg, sausages, coconut, peas, fish, seafood, and beans. A total of 77 out of 102 patients (75.5%) reported some kind of food restriction. 

Also, 69.6% reported often (25.5%) or sometimes (44.1%) sore perianal skin, and 66 patients (65.3%) used ointments, mostly zinc ointment. Thirty-two patients (32%) reported often (6%) or sometimes (26%) pain in the reservoir, and 24% used pain medication. Twenty-five patients (25%) had experienced pouchitis and treated with antibiotics. Thirty-two patients (32%) reported often (6%) or sometimes (26%) pain in their reservoirs. Thirty-six patients (36%) never had pain. Thirteen patients (12.7%) reported voiding problems, mostly hesitation. Two patients reported occasional need of catheterisation. Seventeen patients (16.7%) reported sexual restrictions, mostly men (82%), due to erectile dysfunction. Thirteen (12.7%) reported a better sexual life. Eleven of the men (18.6%) and seven of the women (17.7%) had got children after reservoir construction. All deliveries but one was performed by caesarean section.

Twenty-eight (28.9%) patients reported reduced social activity, mostly due to fatigue, pain, or increased dependency of toilettes. Sixteen patients (16.5%) reported better social life, mostly due to joy of not having ileostomy. Nineteen patients (37.5%) reported reduced physical activity after the reservoir, mostly due to fatigue and dependency of toilette. Twenty-one patients (30%) reported increased physical activity, like bathing, dancing, travel, and training in physical institutes.

Thirty-two patients (32%) reported that life with reservoir was as expected, and 29 patients (29%) thought the result was better. 12 patients (12%) thought it was worse than expected, mostly due to frequent defecations and pain in the reservoir. Three patients (3%) often regretted the reservoir operation, whereas 10 others (10%) sometimes regretted the operation. Only one patient (1%), however, often wanted the ileostomy back, whereas seven reported that they sometimes considered an ileostomy (7.1%). Eighty-three patients (86.4%) reported that life with the reservoir was much better (68 patients (70.8%)) or a little better (15 patients (15.6%)) than life with the ileostomy, whereas nine patients (9.4%) reported that life was a little worse (seven patients (7.3%)) or much worse (two patients (2.1%)) than life with the ileostomy.

**Figure 1:**
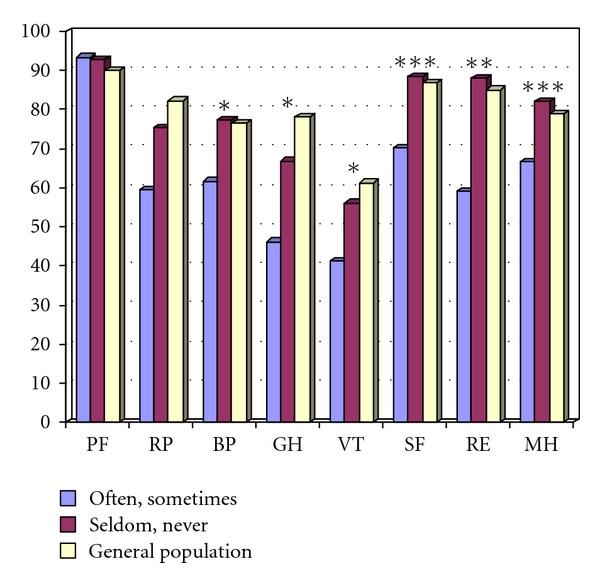
SF-36 scores of 101 patients with J-reservoirs comparing patients who *often or sometimes* regretted the reservoir operation (*n* = 13) with patients who *seldom or never* (*n* = 88) regretted the reservoir operation. Higher scores indicate better function. Values for the general Norwegian population matched on age and gender are also shown. Subscale scores in the Short-Form Health Survey questionnaire (SF-36). Higher scores indicate better function. PF = physical function sum score, RP = role limitations/physical sum score, BP = bodily pain sum score, GH = general health sum score, VT = vitality sum score, SF = social function sum score, RE = role limitation/emotional problems, MH = mental health sum score. Significances are calculated with Student's *t*-test between functional and failed reservoirs: * = *P* < 0.05,  ** = *P* < 0.01, *** = *P* < 0.001.

There was a significant association between the overall satisfaction and several factors of quality of life, as shown in Table 5, as well as the SF-36 scores. With regard to fecal frequency, patients who regretted the operation had significantly higher nighttime faecal frequency at best, average, and worst nights compared to patients that did not regret the operation. Faecal frequencies at daytime periods did not differ between the groups. The 13 patients who regretted the reservoir operation “often or sometimes” had similar SF-36 scores. These scores were significantly lower than the SF-36 scores of patients that “seldom or never” regretted the reservoir operation, which had age- and sex-adjusted scores similar to the general population (Figure 1).

### 4.2. Rectoscopy

Eighty-nine patients (80.2%) attended the followup for clinical examination and rectoscopy. In thirteen patients (14.6%), the perineal skin was red and sore. One (1.1%) patient had a perineal fistula.

All but one patient had normal sphincter tone and squeeze pressure at rectal exploration. 14 patients (15.7%) had slight stenosis at the anastomosis, passable for a finger. Four patients (4.5%) had stenosis not passable for a finger. Patients with anastomotic stenosis had a significantly longer mean residual rectum 1 cm (range: 0–3 cm) versus 2 cm (0–6 cm) than patients without stenosis (*P* = 0.019). In eight patients (9.3%), the reservoir showed inflammation upon rectoscopy. No neoplasm was discovered. The mean length of the reservoir was 13.5 cm (10–20 cm), and the length of residual rectum was 1.2 cm (0–6 cm).

### 4.3. Biopsy

The results of microscopical examination showed variable inflammation in the reservoirs and residual rectum in almost all the patients. The pouch inflammation was global in the pouch, as shown by similarity in biopsies at 5 cm and 10 cm (Table 6). The visual appearance by rectoscopy was not a reliable method to diagnose microscopic inflammation. Of 60 patients with normal visual pouches as determined by rectoscopy, only two had normal mucosa by microscopy. Forty-tree patients had mild inflammation, and 15 had moderate chronic inflammation. However, the five patients with visual inflammation also had microscopically inflammation, mild or moderate. Visual inflammation also seemed more clinical relevant, as these patients had significantly more pain than patients without visible inflammation (*P* = 0.036). All patients with visible inflammation reported some degree of pain in their reservoirs.

There was villous atrophy of various degrees in about 2/3 of the patients, and frank colonisation in about 10% (Table 7). No dysplasia was detected in any patient, neither from the pouch nor from the residual rectal mucosa.

There was no association between observation time and inflammation or villous atrophy. In fact, two of the five patients with colonisation of the mucosa were among patients with the shortest observation time, 612 and 617 days.

### 4.4. Blood Samples

Blood samples were drawn from 76 patients (68.5%) for laboratory tests. Twelve (15.8%), eight (10.7%), and nine (12.1%) of the patients had low levels of haemoglobin, iron, and ferritin, respectively. Eight (10.8%) of the patients had elevated levels of iron-binding capacity. Two patients (2.7%) had low folic acid levels. No cobalamin levels were low. The albumin levels were within normal limits in all patients except two; one patient with too low and one patient with too high levels. The creatinine levels were too low in five patients (6.8%) and elevated in three (4.1%). The levels for bilirubin, ALP, GT, SGOT, and SGPT were elevated in 5.3%, 8.2%, 20.5%, 16.4%, and 6.9%, respectively.

### 4.5. Calprotectin

Faecal calprotectin was measured in 60 patients with a mean value of 331.7 mg/kg (5–1275 mg/kg). There was an association between calprotectin levels and signs of macroscopically inflammation in the reservoir. Patients with macroscopic inflammation in their reservoirs had significantly higher calprotectin levels (826 mg/kg (137–1275 mg/kg) than patients with macroscopically normal reservoirs (289 mg/kg (1–1120 mg/kg) (*P* = 0.019). There was no association between microscopic inflammation and calprotectin levels. Patients with anastomosis stenosis had the same calprotectin levels as patients without stenosis. 

There was also an association between the level of calprotectin and previous episodes of pouchitis. Patients with previous pouchitis had mean calprotectin levels of 510 mg/kg (47–1275 mg/kg) versus levels of 257 mg/kg (5–1067 mg/kg) in patients that had not experienced pouchitis (*P* = 0.007).

### 4.6. HP Status

The rapid urease test (RUT) for detection of *Helicobacter pylori* in tissue samples of the pouch mucosa was performed in 71 patients. In 30 patients (42.3%), the test was positive. In 41 patients (57.7%), the test was negative. The immunochromatographic test (ImmunoCard HpSA STAT) of stool samples showed quite different results. Only 36 patients had enough stool sample for reliable tests. In these patients, the test was positive in four (11.1%) patients, and negative in 32 patients (88.9%). There was no association between the two tests, as two of the ImmunoCard-positive patients had negative RUT-tests, and eleven of the ImmunoCard-negative patients had positive RUT-tests. 

There was no association between the tests and previous pouchitis, macroscopic, microscopic inflammation, or villous atrophy in the pouch.

## 5. Discussion

The present study showed a wide spectrum of functional results in patients with the J-reservoir. Most patients were satisfied, and many reported improved quality of life after the reservoir operation. 87.1% of the patients seldom or never regretted the operation and had SF-36 scores similar to the general population. The study showed, however, that most of the patients had some kind of restriction in their daily life; in fact, 93.1% of the patients reported some kind of restriction in their work, or social, physical, or sexual life. Only seven patients (6.9%) reported no restrictions at all. Most patients reported some kind of dietary problems, but the type of food that caused their problems was quite inhomogeneous, and different food types had obviously different effect on different patients. This is in line with previous reports of dietary restrictions of up to 95% of patients after reservoir construction [3]. In spite of this, only 13 patients (12.9%) stated that they regretted the reservoir operation often or sometimes. This overall impression was reflected in the SF-36 scores. Patients who regretted the operation often or sometimes had about the same SF-36 scores, which were significantly lower than patients who regretted the operation “seldom or never.” It was in particular the social, emotional, and mental dimensions as well as the general health and vitality scores that were affected. 

With regard to factors of importance for the less favourable results, faecal frequency clearly relates to quality of life. Patients with high faecal frequencies rates had more restrictions in their daily life than patients with low faecal frequencies. This was also reflected in lower SF-36 scores. We looked for organic causes for these functional results. The only significant finding was that faecal frequency was related to macroscopic inflammation in the reservoir, which was related to more pain in the reservoir and higher levels of calprotectin than patients with reservoirs without macroscopically inflammation. Thus, high daytime faecal frequency and pouchitis are determinants of an unfavourable functional outcome. This could, however, explain only some of the cases of frequent defecation. Also high faecal frequency was not the only determinant for a less successful result, as most patients in the group with high faecal frequency rate never regretted the reservoir operation. 

Our results of faecal frequency, faecal control, and incidence of pouchitis were similar to others [2, 5]. The patients with a history of pouchitis had higher calprotectin levels than patients with no history of pouchitis. It has previously been shown that pouchitis is associated with elevated faecal calprotectin levels, which correlates with the level of mature granulocytes and activated macrophages in the lamina propria of the bowel wall [15, 16] and indicates that the ileal pouch patients with previous history of pouchitis have a more reactive pouch than patients without pouchitis. 

Even if development of cancer in the pouch is a feared complication, dysplastic transformation is considered a rare phenomenon [17]. However, it does occur, and several cases have been reported. Most of these cancers probably arise from the residual colon mucosa [18], but primary cancers arising from the ileal pouch have been described [9]. These authors suggest a malignant transformation of the ileal pouch mucosa based on a passage from chronic inflammation with chronic atrophic pouchitis to dysplastic epithelium and to cancer. In the present study, no dysplasia or cancer was discovered in the pouch or in the residual colon mucosa. Eight patients had visible inflammation. However, all patients except two had mild or moderate chronic inflammation as determined by microscopical investigation of biopsies. 66% of the patients showed villous atrophy, and most patients had slight or moderate atrophy. Only 11 patients had severe atrophy, five of them had colon-like mucosa. This is in accordance with Börjesson et al., who described persisting severe mucosal atrophy in a minor proportion of the patients after a median followup of 16 years [18]. There was no relation between observation time and degree of atrophy. Many patients had reservoirs for a long time without signs of atrophy, and in some the atrophy was observed after a short period. In two of the five patients with colonisation, these changes were seen in reservoirs of the shortest observation periods, 612 and 617 days. 

The two tests of *Helicobacter pylori* showed quite different results, as HP was detected in 42.3% of the urease tests and 11.1% of the stool samples. There was association neither between the two tests, nor between HP status and previous history of pouchitis, macroscopic, microscopic inflammation, or villous atrophy in the pouch. The reason for the discrepancy between the tests remains uncertain. Other causes than HP infection must exist for inflammation and atrophy in the reservoir.

Of 125 patients treated with J-reservoir and IPAA at our institution, one reservoir was removed due to postoperative complications, and 13 patients (10.5%) had reservoir failure in the followup time. In addition, 13 patients with functional reservoirs had unfavourable functional results with decreased quality of life scores as shown in this study. Thus, a total of 27 patients (21.6%) had an unfavourable result, whereas 78.4% were satisfied. The results of other published series differ considerably, with failure rates from 3.4% in the best reports to 8–11% in others [6, 7, 19]. Failure rates in these reports refer to the removal or defunctioning of the reservoirs. Quality of life measures in patients with functional reservoirs also vary. Several authors report excellent long-term results, with good functional results and overall satisfaction rates of 95% [2, 5, 20]. The reasons for these apparent discrepancies may be patient selection, attention to surgical details, and postoperative followup, as is reported from the Cleveland Clinic [19], but observation time and type of questions asked in the quality of life questionnaire may also be of importance. A meta-analysis of 9317 patients showed a pouch failure rate of 6.8% rising to 8.5% in case followup of more than 60 months. Severe, mild, and urge faecal incontinence occurred in 3.7%, 17%, and 7.3%. The authors stated that the current techniques for restorative surgery after proctocolectomy are associated with nonnegligible complication rates and leave room for improvement and continuation of development of alternative procedures [8].

## 6. Conclusions

Proctocolectomy with IPAA offers a possibility to lead a normal life for most patients when medical treatment fails. The results, however, constitute a functional spectrum from reservoir failures in need of resection or defunctioning, functional reservoirs with several problems in the daily life to excellent results. Aside from postoperative pelvic sepsis and anastomotic leaks that often lead to reservoir failure, the results are often unpredictable. High faecal frequency rates, visible inflammation in the reservoir, and episodes of pouchitis are associated with less favourable function, but it is not possible to predict who will have these complications. A number of patients will develop problems without any detectable reason. Even after long-term followup, dysplasia was not seen, which is reassuring for the patients and the surgeons.

## Figures and Tables

**Table 1:** Characteristics of 111 patients with functional J-reservoirs after long-term followup.

Gender (men/women)	65 (58.6%)/46 (41.6%)
Age at start of disease	28.0 years (3–60 years)
Age at reservoir construction	35.9 years (9–69 years)
Age at followup	42.6 years (17–72 years)
Observation time: time	6.8 years (0.8–15.3 years)
with functional reservoir	

**Table 2:** Elements of quality of life at followup in 103 patients with functional J-reservoirs.

	*n* (%)
Average stool frequency in *daytime* (median (range))	
Best time periods	4 (1–10)
Average time periods	6 (2–13)
Worst time periods	10 (4–30)
Average stool frequency at *night* (median (range))	
Best time periods	0 (0–3)
Average time periods	1 (0–6)
Worst time periods	3 (0–12)
Patients at work: 100% work/reduced work	68 (66.7%)/13 (12.8%)
Faecal control always/almost always	38 (37.3%)/45 (44.1%)
Faecal incontinence often/sometimes	6 (5.9%)/13 (12.7%)
Treated for pouchites	25 (25%)
Dietary restrictions	76 (75.2%)
Sexual life better/worse (restriction)	13 (12.7%)/17 (16.7%)
Social activity better/worse (restriction)	16 (16.6%)/28 (28.9%)
Physical activity better/worse (restriction)	21 (30.0%)/19 (27.5%)
Regretted reservoir often/sometimes	3 (3.0%)/10 (9.9%)
Life compared to ileostomy: much better/a little better	68 (70.8%)/15 (15.6%)
Life compared to ileostomy: a little worse/much worse	7 (7.3%)/2 (2.1%)

**Table 3:** Influence of faecal frequency on SF-36 subscores during best, average, and worst day and night periods. The SF-36 subscores for patients with low, medium, and high fecal frequencies during these periods are compared.

Time	Quality of days	Category of number of defecations	PF	RP	BP	GH	VT	SF	RE	MH
	Best time periods (*n* = 92)	Low: 1–3 (*n* = 32) Medium: 4 (*n* = 33) High: 5–10 (*n* = 27)	ns	0.009	ns	0.000	ns	ns	ns	ns
Day	Average periods (*n* = 100)	Low: 2–4 (*n* = 27) Medium: 5–6 (*n* = 43) High: 7–13 (*n* = 30)	0.004	0.006	0.013	0.002	0.009	ns	ns	ns
	Worst periods (*n* = 92)	Low: 4–7 (*n* = 20) Medium: 8–10 (*n* = 45) High: 11–30 (*n* = 27)	0.019	0.009	0.005	0.006	0.004	0.009	0.033	ns

	Best time periods (*n* = 94)	Low: 0 (*n* = 58) High: 1–3 (*n* = 36)	0.024	0.038	ns	0.037	0.006	ns	ns	ns
Night	Average periods (*n* = 101)	Low: 0 (*n* = 17) Medium: 1 (*n* = 48) High: 2–6 (*n* = 36)	ns	0.037	ns	ns	ns	ns	ns	ns
	Worst periods (*n* = 92)	Low: 0–2 (*n* = 31) Medium: 3–4 (*n* = 37) High: 5–12 (*n* = 24)	ns	ns	ns	ns	ns	0.010	ns	ns

PF = physical function sum score; RP = role limitations/physical sum score; BP = bodily pain sum score; GH = general health sum score; VT = vitality sum score; SF = social function sum score; RE = role limitation/emotional problems; MH = mental health sum score. Significant differences as tested by one-way ANOVA are shown.

**Table 4:** Influence of *average daytime stoolfrequency* on factors of importance for quality of life in 100 patients with low (2–4 per day), medium (5–6 per day), and high (7–13 per day) faecal frequency.

	Low	Medium	High	
	(2–4 pr. day)	(5–6 pr. day)	(7–13 pr. day)	*P* ^(a)^
	*n* = 27 %	*n* = 43 %	*n* = 30 %	
Problems in work (often/sometimes)	26	26	63	0.013
Sore perineum (often/sometimes)	59	70	83	0.000
Pain in reservoir (often/sometimes)	22	26	52	0.045
Medication for pain (often/sometimes)	7	2	21	0.006
Use of diaper (often/sometimes)	22	16	40	0.051
Avoid food due to loose stool	48	50	83	0.006
Avoid food due to other problems	23	40	71	0.001
Social life better/worse	17/13	20/22	13/50	0.026
Sexual life better/worse	19/15	14/14	7/23	0.485
Reservoir worse than expected	8	10	20	0.484
Want ileostomy back often/sometimes)	0	5	20	0.130
Life compared to ileostomy much better	81	75	54	0.134
Regretted reservoir operation often/sometimes)	11	7	23	0.443

^(a)^ *P* value from Pearson's Chi-square test.

**Table 5:** Factors which influence the overall functional result of the J-reservoir.

	Regretted reservoir operation		
	Often/sometimes	Seldom/never	*P*
	(*n* = 13) %	(*n* = 88) %	
Problems in work (often/sometimes)	62	33	0.034
Changed profession after reservoir	38	12	0.013
Inflamed reservoir/injected	45	3	0.000
Pain in reservoir (often/sometimes)	58	28	0.018
Medication for pain (often/sometimes)	33	6	0.017
Treated for pouchitis in the reservoir	54	24	0.028
Number of pouchitis	5 ± 10	1 ± 3	0.002^#^
Fecal incontinence (often/sometimes)	46	15	0.014
Use of diaper (often/sometimes)	38	24	0.024
Voiding problems still	31	10	0.003
Physical activity increased/reduced	10/70	33/20	0.001
Social life better/worse	0/69	19/23	0.002
Sexual life better/worse	0/54	15/11	0.001
Avoid food due to loose stool	77	57	0.182
Avoid food due to other problems	42	45	0.849
Reservoir worse than expected	62	5	<0.001
Want ileostomy back (often/sometimes)	67	0	<0.001
Life compared to ileostomy (much better)	27	76	<0.001
Sufficient preoperative information	31	69	0.025

Significances tested by Student's *t*-test ^(#)^ and Pearson Chi-square test.

**Table 6:** Degree of inflammation in biopsies from the J-reservoir at 5 cm and 10 cm from the rectal anastomoses and from the residual rectum.

	Biopsies from the J-pouch		Rectum
	5 cm	10 cm	Rectum
	(*n* = 65)	(*n* = 69)	(*n* = 64)
	*n* (%)	*n* (%)	*n* (%)
No inflammation	2 (3.1%)	3 (4.3%)	6 (9.4%)
Mild chronic	46 (70.7%)	51 (73.9%)	40 (62.4%)
inflammation			
Moderate chronic	17 (26.2%)	14 (20.4%)	12 (18.8%)
inflammation			
Severe chronic	—	—	3 (4.7%)
inflammation			
Mild acute	—	1 (1.4%)	3 (4.7%)

**Table 7:** Villous atrophy in biopsies from the J-reservoir at 5 cm and 10 cm from the rectal anastomoses.

	Biopsies from the J-pouch	
	5 cm	10 cm
	(*n* = 54)	(*n* = 56)
	*n* (%)	*n* (%)
No villous atrophy	18 (33.3%)	21 (37.5%)
Mild villous atrophy	27 (50.0%)	24 (42.9%)
Moderate villous atrophy	4 (7.4%)	6 (10.7%)
Colonisation of the pouch mucosa	5 (9.3%)	5 (8.9%)
